# Early Gastrointestinal Progression to Immunotherapy in Lung Cancer: A Report of Two Cases

**DOI:** 10.1155/2021/6692538

**Published:** 2021-02-26

**Authors:** Federica Martorana, Katia Lanzafame, Giuliana Pavone, Lucia Motta, Gianmarco Motta, Nicola Inzerilli, Rosaria Carciotto, Giada Maria Vecchio, Antonino Maria Zanghì, Héctor Josè Soto Parra, Gaetano Magro, Paolo Vigneri

**Affiliations:** ^1^Dept. of Clinical and Experimental Medicine, University of Catania-Catania, Via Santa Sofia, 78-95123 Catania, Sicily, Italy; ^2^Division of Medical Oncology-A.O.U. Policlinico G. Rodolico-San Marco-Catania, Via Santa Sofia, 78-95123 Catania, Sicily, Italy; ^3^Center of Experimental Oncology and Hematology-A.O.U. Policlinico G. Rodolico-San Marco-Catania, Via Santa Sofia, 78-95123 Catania, Sicily, Italy; ^4^Dept. of Medical and Surgical Sciences and Advanced Technology G. F. Ingrassia-A.O.U. Policlinico G. Rodolico-San Marco-Catania, Via Santa Sofia 87, 95123 Catania, Sicily, Italy

## Abstract

Intestinal and pancreatic metastases are rare and often challenging to recognize and manage. Lung cancer patients with enteric involvement usually display poor outcomes. Hyperprogression to immunotherapy represents a concern, even though there is currently no agreement on its exact definition. Gastrointestinal hyperprogression to immune checkpoint inhibitors has not been described so far. In these cases, distinguishing disease-related symptoms from immune-related adverse events may represent a diagnostic conundrum. Here, we report two cases of non-small-cell lung cancer experiencing a rapid pancreatic and colic progression to immunotherapy, respectively. While further investigations to identify biomarkers associated with hyperprogression are warranted, clinicians should be aware of the potential unusual clinical presentations of this phenomenon.

## 1. Introduction

Lung cancer commonly metastasizes to the bone, contralateral lung, liver, adrenal glands, brain, and lymph nodes, but less frequently it spreads to other anatomic sites, including the gastrointestinal system [1, 2]. Indeed, enteric metastases have been infrequently described in patients with non-small-cell lung cancer (NSCLC). Clinical presentation can be either abrupt with bleeding, bowel obstruction, or perforation, requiring intensive care in a hospital setting, or subacute, with abdominal pain, progressive anaemia, or jaundice. Unfortunately, patients with gastrointestinal metastases display a dismal prognosis regardless of their clinical presentation [3–5]. While immunotherapy has significantly improved the outcomes of stage IV NSCLC, a subset of patients receiving immune checkpoint inhibitors experience rapid disease evolution during treatment, a phenomenon known as hyperprogression (HP) [6, 7]. To the best of our knowledge, no data are available about the occurrence of gastrointestinal HP to immunotherapy in lung cancer.

Here, we report two cases of NSCLC with a rapid and unusual pancreatic and intestinal progression during treatment with the anti-Programmed Death-1 (PD-1) monoclonal antibody nivolumab.

## 2. Cases Presentation

### 2.1. Case#1

**Figure 1:**
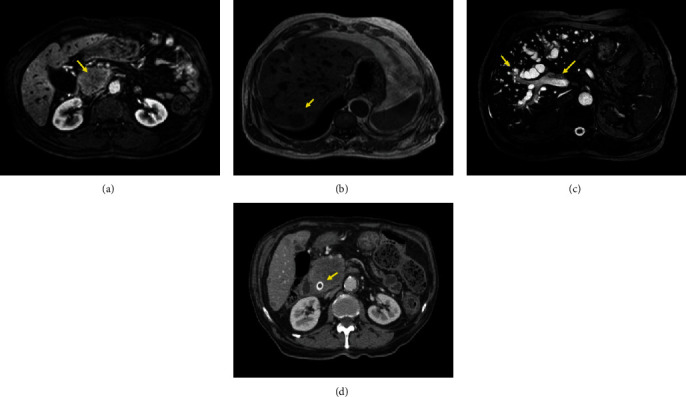
MR imaging of the pancreatic head mass (a) of the larger liver metastasis (b) and of the biliary dilatation (c). CT imaging of the biliary drainage (d).

In December 2015, a 68-year-old Caucasian man with a 55 pack-years smoking history, chronic obstructive pulmonary disease, type 2 diabetes, hypercholesterolemia, and hypertension presented to the Emergency Room because of persistent cough and haemoptysis. A whole body Computed Tomography (CT) scan revealed a 60 mm mass in the left lung along with several satellite nodules in the same lobe. Both the CT and an ensuing Positron Emission Tomography (PET) scan excluded the presence of distant metastases. Thus, in February 2016, he underwent a left pneumonectomy with lymph nodal dissection. The pathology report indicated the presence of a poorly differentiated squamous cell carcinoma, pT3 (main diameter 60 mm) N0 (0 out of 24) M0 (stage II according to the UICC/AJCC TNM 7th edition). From March to July 2016, he received four cycles of adjuvant cisplatin plus vinorelbine, but one month after completing the last chemotherapy infusion, both CT and PET scans showed hepatic and para-aortic nodal relapse that were confirmed by a liver biopsy. Programmed Death-Ligand 1 (PD-L1) immunohistochemical expression, assessed on the metastatic tissue, was below 1%. Given the short interval between the end of adjuvant chemotherapy and the disease relapse, in September 2016 the patient started a second line treatment with nivolumab 3 mg/kg every two weeks. After the third administration of immunotherapy, he developed progressive jaundice, fever, nausea, and vomiting along with a rise in bilirubin, alkaline phosphatase, and glutamyl-transpeptidase. Because of these symptoms, initially attributed to an immune-related adverse events, the patient was admitted to the oncology ward where he received high-dose corticosteroids (prednisone 1 mg/kg intravenously). However, in the absence of any meaningful clinical benefit after 48 hours, he performed a Magnetic Resonance Imaging (MRI) of the abdomen that showed a bulky lesion of the pancreatic head, partially infiltrating the duodenal wall and causing visible distension of both the Wirsung and the intrahepatic biliary ducts. Moreover, multiple hepatic and nodal metastases emerged (Figure 1). Given these findings, the patient underwent percutaneous trans-hepatic cholangiography with biliary drainage, stenting, and cytological sampling, the latter consistent with lung cancer metastasis. Once bilirubin levels normalized, he began third line chemotherapy with weekly paclitaxel. However, in the following six months his clinical conditions progressively deteriorated, and in June 2017, he developed persistent fever, abdominal pain, anorexia, and weight loss. He therefore suspended active treatment and eventually died in August 2017.

### 2.2. Case#2

**Figure 2:**
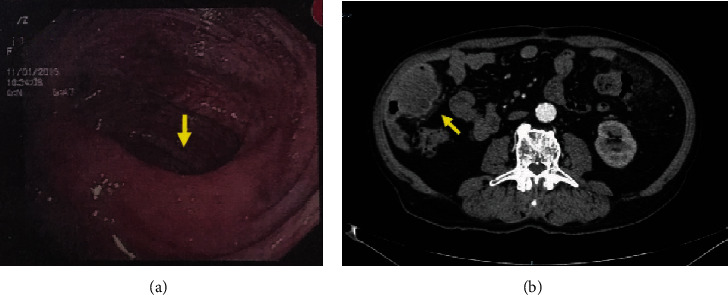
Endoscopic imaging of lung adenocarcinoma metastasis in the splenic flexure and (a) CT imaging of left colonic wall thickening (b).

**Figure 3:**
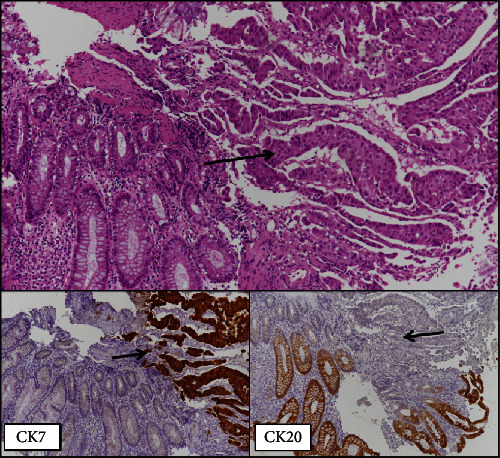
Intestinal biopsy: normal colonic mucosa infiltrated by solid-pseudoglandolar carcinoma, positive for CK7 and negative for intestinal differentiation markers, such as CK20 and CDX2 (arrows). Lung adenocarcinomas with prevalent solid pattern may be negative for TTF-1 but often positive for CK7. On the basis of the morpho-immunohistochemical data and clinical history, the diagnosis of intestinal metastasis of lung adenocarcinoma was made.

**Figure 4:**
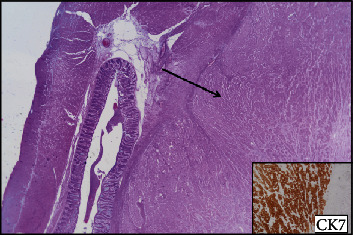
Surgical findings: the diagnosis of lung adenocarcinoma metastasis was confirmed after surgical resection. The figure shows intramural CK7 positive neoplasia, with a normal underlying colonic mucosa.

In November 2016, a 69-year-old Caucasian male came to our attention because of the acute onset of dyspnoea and haemoptysis. He was a 35-pack-year former smoker since 2010, and his previous medical history was notable for myocardial infarction, diabetes mellitus, bilateral glaucoma, and benign prostatic hyperplasia. A CT scan showed a 40 × 33 mm mass in the right lung invading the main bronchus, with increased metabolic activity (Standardized Uptake Value (SUV) 41) at the PET scan. An ensuing bronchoscopy indicated the presence of a poorly differentiated lung adenocarcinoma for which he received four cycles of neo-adjuvant chemotherapy with cisplatin and vinorelbine, achieving a satisfactory dimensional and metabolic response. Thus, in April 2017, he underwent a right upper lobectomy with mediastinal node dissection. The pathologist's assessment revealed a 30 mm poorly differentiated adenocarcinoma of the lung, Cytokeratin 7, and Thyroid Transcription Factor 1 (TTF1) positive, with involvement of the main bronchus (pT2b) and no nodal metastases (0 out of 21; stage IIB according to the UICC/AJCC TNM 7th edition). Five months after surgery the patient noticed a rapidly growing subcutaneous lump in his right thigh and another one in his left axilla, both excised and histologically proven to be lung adenocarcinoma metastases. No Epidermal Growth Factor Receptor (*EGFR*) mutations or Anaplastic Lymphoma Kinase (*ALK*) translocations were detected, and immunohistochemical staining for PD-L1 was below 1%. Hence, in December 2017, he began first line chemotherapy with carboplatin and pemetrexed, experiencing disease stabilization, followed by pemetrexed maintenance until April 2018, when a CT scan showed an abdominal nodal progression. At this time, the patient received second line treatment with nivolumab 240 mg every two weeks, from May to August 2018. However, throughout these months, he experienced progressively worsening fatigue and developed G1 anaemia. After the 6th nivolumab cycle, a CT scan revealed a new cerebellar lesion, confirmed by MRI and treated with stereotactic radiation therapy. The CT exam also documented extensive nodal progression and a left colonic wall thickening, further investigated with a colonoscopy which revealed an ulcerated lesion in the splenic flexure histologically consistent with a lung adenocarcinoma metastasis (Figures 2 and 3). Thus, in September 2018, he underwent palliative segmental resection of the left colon, which confirmed the diagnosis of intestinal metastasis of lung adenocarcinoma (Figure 4). Upon recovery, he began third line chemotherapy with weekly paclitaxel of which he received only two cycles, as he developed both G2 anaemia and melena. A new colonoscopy showed cancer relapse at the site of the previous surgery, whereas a CT scan performed in January 2019 revealed nodal and hepatic progression, along with a femoral vein thrombosis. Because of the latter finding, interventional radiologists positioned an inferior vena cava filter. The patient continued best supportive care and died in August 2019.

## 3. Discussion

We describe here two cases of metastatic NSCLC with unusual early gastrointestinal progression to the immune checkpoint inhibitor nivolumab. Both patients were heavy smokers (i.e., >30 pack-years) presenting with a squamous cell carcinoma or an adenocarcinoma, the histological variants most frequently associated with pancreatic and intestinal involvement, respectively [3, 4, 8]. However, no data are currently available concerning the *EGFR*, *ALK*, and PD-L1 status of these tumours. Consistent with preexisting evidence, clinical manifestations of gastrointestinal metastases were severe and led to treatment discontinuation, heavily worsening the patients' quality of life [5]. Moreover, symptoms were difficult to recognize, and even after radiological diagnosis, a biopsy was mandatory to rule out a second synchronous cancer. Although our patients received additional chemotherapy after discontinuing nivolumab, they lacked any consistent benefit from the subsequent treatment (weekly paclitaxel). However, our patients experienced a 9-month and a 12-month survival from the detection of the enteric lesions, respectively. Hence, their survival is significantly longer than the one reported in the literature (2.8 months) [4, 5].

To the best of our knowledge, this is the first report of gastrointestinal hyperprogression to immunotherapy in lung cancer. Of note, Miyazawa and colleagues reported two cases of metastatic melanoma spreading to the small bowel and the colon during nivolumab treatment [9]. Due to the rapid and extensive disease progression that our patients experienced, we deemed them “hyper-progressors.” Although several studies recently provided an overview about HP, this phenomenon is still matter of debate [10–12]. However, preliminary evidences are emerging about potential molecular biomarkers useful in the timely identification of hyperprogressing patients [11, 13–15]. It is crucial to promptly and correctly recognize NSCLC gastrointestinal progression as this atypical metastatic spread, usually associated with subtle and equivocal symptoms, may result in diagnostic delay. In addition, if an intestinal progression occurs during immunotherapy, its clinical identification can be extremely challenging as it may be potentially mistaken for an immune-related complication. The present report emphasizes the possibility of an unusual enteric metastatic disease in patients affected by NSCLC treated with immune checkpoint inhibitors. As an increasing number of patients will receive anti-PD-1 or anti-PD-L1 antibodies, a concerted effort is needed in order to identify biomarkers predictive of HP.

## Data Availability

The underlying data supporting the results of the study can be found in the manuscript.
